# Perinatal Mortality Analysis in Espírito Santo, Brazil, 2008 to 2017

**DOI:** 10.3390/ijerph182111671

**Published:** 2021-11-06

**Authors:** Italla Maria Pinheiro Bezerra, José Lucas Souza Ramos, Micael Colodetti Pianissola, Fernando Adami, João Batista Francalino da Rocha, Mariane Albuquerque Lima Ribeiro, Magda Ribeiro de Castro, Juliana da Fonsêca Bezerra, Fabiana Rosa Neves Smiderle, Luiz Vinicius de Alcantara Sousa, Carlos Eduardo Siqueira, Luiz Carlos de Abreu

**Affiliations:** 1 Departamento de Pós-Graduação em Políticas Públicas e Desenvolvimento Local, Escola Superior de Ciências da Santa Casa de Misericórdia de Vitória (EMESCAM), Vitória 29027502, Brazil; 2 Departamento de Enfermagem, Laboratório de Escrita Científica, Escola Superior de Ciências da Santa Casa de Misericórdia de Vitória (EMESCAM), Vitória 29027502, Brazil; joselucasenfermeiro@gmail.com (J.L.S.R.); micaelpianissola@gmail.com (M.C.P.); fabiana.neves@emescam.br (F.R.N.S.); 3 Laboratório de Epidemiologia do Centro Universitário ABC (FMABC), Santo André 09060590, Brazil; adamifernando@uol.com.br; 4 Ciências da Saúde no Centro Universitário ABC (FMABC), Santo André 09060870, Brazil; joao.rocha@aluno.fmabc.net (J.B.F.d.R.); mariane.ribeiro@ufac.br (M.A.L.R.); 5 Centro de Ciências da Saúde e do Desporto (CCSD), Universidade Federal do Acre (UFAC), Rio Branco 69920900, Brazil; 6 Departamento de Enfermagem da Universidade Federal do Espírito Santo (UFES), Vitória 29075910, Brazil; magda.soares@ufes.br; 7 Departamento de Enfermagem Materno Infantil (DEMI) da Universidade Federal do Rio de Janeiro (UFRJ), Rio de Janeiro 21941901, Brazil; julianabezerra@eean.ufrj.br; 8 Departamento de Saúde da Coletividade do Centro Universitário FMABC, Santo André 09060870, Brazil; viniciusdealcantaras@gmail.com; 9 Environment and Public Health, School for the Environment, Transnational Brazilian Project, The Mauricio Gastón Institute for Latino Community Development and Public Policy, UMass Boston, Boston, MA 02125, USA; carlos.siqueira@umb.edu; 10 Departamento de Educação Integrada em Saúde na Universidade Federal do Espírito Santo (UFES), Vitória 29027502, Brazil; luiz.abreu@ufes.br

**Keywords:** perinatal mortality, fetal death, early neonatal mortality, infant mortality, maternal and child health

## Abstract

This is an ecological and time-series study using secondary data on perinatal mortality and its components from 2008 to 2017 in Espírito Santo, Brazil. The data were collected from the Mortality Information System (SIM) and Live Births Information System (SINASC) of the Unified Health System Informatics Department (DATASUS) in June 2019. The perinatal mortality rate (×1000 total births) was calculated. Time series were constructed from the perinatal mortality rate for the regions and Espírito Santo. To analyze the trend, the Prais–Winsten model was used. From 2008 to 2017 there were 8132 perinatal deaths (4939 fetal and 3193 early neonatal) out of a total of 542,802 births, a perinatal mortality rate of 15.0/1000 total births. The fetal/early neonatal ratio was 1.5:1, with a strong positive correlation early neonatal mortality rate, perinatal mortality rate, r (9) = 0.8893, with a significance level of *p* = 0.000574. The presence of differences in trends by health region was observed. Risk factors that stood out were as follows: mother’s age ranging between 10 and 19 or 40 and 49 years old, with no education, a gestational age between 22 and 36 weeks, triple and double pregnancy, and a birth weight below 2499 g. Among the causes of death, 49.70% of deaths were concentrated in category of the tenth edition of the International Classification of Diseases, fetuses and newborns affected by maternal factors and complications of pregnancy, labor, and delivery (P00–P04), and 11.03% were in the category of intrauterine hypoxia and birth asphyxia (P20–P21), both related to proper care during pregnancy and childbirth. We observed a slow reduction in the perinatal mortality rate in the state of Espírito Santo from 2008 to 2017.

## 1. Introduction

Perinatal mortality is characterized by the number of fetal deaths from 22 complete weeks of pregnancy to the sixth full day of life, reflecting access to health services and the quality of prenatal, delivery, and newborn care [1,2]. It is estimated that 4.9 million perinatal deaths occur worldwide, with 2 million of these being fetal and 2.9 being early neonatal [3,4], and approximately 98% of all neonatal and perinatal deaths occur in low- and middle-income countries [5].

In Brazil, most are avoidable and currently invisible [6], with a total of 53,170 deaths [7]. In 2010, perinatal mortality in Brazil declined compared to 2000, reaching 21.5 deaths/1000 total births in 2010, while in 2000 the rate was 26.6/1000, representing a reduction of 16.1%. It was higher in the North and Northeast regions with rates of 19.5/1000 and 22.1/1000, respectively, while the South and Southeast had rates of 14/1000 and 16/1000, respectively [7].

In 2014, the World Health Organization (WHO) launched an action plan to reduce preventable deaths and fetal deaths by 2035 [4]. Target 3.2 of the Sustainable Development Goals (SDGs) is to end preventable deaths of newborns and children under five, and to reduce neonatal mortality in all countries; however, they do not prioritize the monitoring of still-births [8,9]. With this being the case, countries that have statistical information systems with good coverage and reliable data enable the monitoring and evaluation of the perinatal epidemiological situation, as well as the planning of interventions for maternal and newborn health [10].

The determinants of perinatal mortality are characterized as multiple and complex, and are related to the interaction of biopsychosocial, healthcare, and economic variables, which vary according to different contexts, times, and spaces. Biological variables are related to the mother and newborn and are the direct causes of neonatal deaths. Access to health services and the quality of care provided in prenatal care, the delivery room, and postnatal care for the newborn are healthcare variables. The socioeconomic variables indicate the conditions in which the mother lives, which are capable of influencing some effects of biological variables and hindering access to adequate care during pregnancy and birth [11]. Many perinatal deaths are concentrated in less-developed regions, where the decline of these deaths occurs slowly [12,13].

Several studies have identified the following risk factors that are associated with perinatal death. Risk factors included: a low level of maternal education [14], single women [14], overweight or obese mothers [14,15], alcohol abuse during pregnancy [14], previous preterm birth [14,16], spontaneous or induced abortions [14,16], antepartum hemorrhage [14,16], fetal growth retardation [14], infections/sepsis [17,18], pre-pregnancy-induced hypertensive disorders [18], and severe anemia [18].

Most causes of perinatal mortality are preventable [5,19]; the possible actions that would reduce perinatal mortality address prematurity- (58%), intrapartum- (79%), and infection-related deaths (84%) [5]. Better access to health services and a higher quality of care provided in the preconception, prenatal, intrapartum, and postnatal periods by 2025 could prevent 71% of neonatal deaths, 33% of stillbirths, and 54% of maternal deaths per year [5,20], which led the authors to focus on perinatal mortality in the state of Espírito Santo. The novelty of this study is that it reveals the results of public policies that were proposed and implemented that aimed at comprehensive care for the health of women and children, as the state was the first, among the Brazilian states, to contract the Rede Cegonha, a strategy of the Ministry of Health that aims to implement a care network to ensure women the right to reproductive planning and humanized care during pregnancy, childbirth, and puerperium, as well as ensuring children the right to a safe birth and healthy development.

Thus, it is relevant to carry out a more detailed analysis using perinatal mortality indicators in the state of Espírito Santo to support the planning and organization of preventive measures aimed at priority areas according to the distribution of risk, in addition to contributing to the discussion about social inequalities and vulnerabilities. From this perspective, the objective is to analyze perinatal mortality and its fetal and early neonatal components in the state of Espírito Santo from 2008 to 2017.

## 2. Materials and Methods

### 2.1. Study Design

This is an ecological study of time series based on secondary data on perinatal deaths and their fetal and early neonatal components, with the state of Espírito Santo as the unit of analysis in the period from 2008 to 2017. This work is part of the Research and Innovation Support Foundation of Espírito Santo (FAPES), under the notice FAPES/CNPq/Decit-SCTIE-MS/SESA No. 03/2018—PPSUS.

### 2.2. Study Location

This study was conducted in the state of Espírito Santo, located in the Southeastern region of Brazil, bordering the state of Bahia to the north, the state of Minas Gerais to the west, the state of Rio de Janeiro to the south, and the Atlantic Ocean to the east. It has a territorial area of 46,074.477 km^2^, an estimated population of 4,064,052 people, a demographic density of 76.25 inhabitants/km^2^, an HDI of 0.74, a humid coastal tropical climate influenced by the Atlantic tropical air mass, an average temperature between 22 and 24 °C, and annual rainfall of between 1000 and 1500 mm.

### 2.3. Study Population

The present study included perinatal deaths and their fetal and early neonatal components in the state of Espírito Santo, Brazil, between 2008 and 2017. Data on perinatal deaths of residents that occurred in the state of Espírito Santo between 2008 and 2017 were obtained by combining the data on fetal deaths with those on early neonatal deaths, as there is no tabulation option for perinatal deaths in the database of the Information Technology Department of the Unified Health System (DATASUS). 

### 2.4. Data Collection

Data were retrieved from the Mortality Information System (SIM) and the Live Birth Information System (SINASC) through the DATASUS database, accessed on 20 June 2019 at https://datasus.saude.gov.br/.

The strategy used for data access, tabulation, and retrieval was as follows: access to https://datasus.saude.gov.br/ > access to information > TAB-NET health information > vital statistics > live births—since 1994 (data collection on live births) > live births > Espírito Santo > in the tab environment, the options were selected and blocked in the fields of column: year of birth; content: birth by mother’s residence; available periods: from 2008 to 2017; and in the “line” field: year of birth. For the collection of data on fetal mortality, on the DATASUS website, from the vital statistics option, mortality was selected—since 1996 by the ICD-10 > fetal deaths > Espírito Santo > in the tabulation environment, the selection and blocking of the options in the fields of column: year of death; content: deaths by mother’s residence; available periods: 2008 to 2017; available selections: duration of pregnancy from 22 weeks to 42 and more + ignored; and in the “row” option, each time the choice of variables. Additionally, for data on early neonatal mortality, the extraction procedure was as follows: in DATASUS, in the vital statistics option, followed > mortality—since 1996 by ICD-10 > infant mortality > Espírito Santo > in the tabulation environment, the selection and blocking of options were made in the fields of column: year of death; content: deaths by residence; the period available: from 2008 to 2017; available selections: age group 1 (0 to 6 days); and in the “row” field, each time the study variables were tabulated.

### 2.5. Study Variables

The dependent variable is the number of perinatal deaths. Neonatal deaths were classified as death cases after 22 complete weeks of gestation (154 days), added to those of unknown gestational age plus deaths occurring up to the 6th complete day of life, per thousand total births (fetal deaths plus live births).

The study’s independent variables are the rates of fetal death and early neonatal death; temporal and geographic definition of deaths: years, health regions, and place of occurrence; characterization of deaths: maternal age, education and marital status, duration of pregnancy, type of pregnancy and delivery, sex, race/skin color, and birth weight of the fetus and newborn; and ICD-10 cause classification: chapter and category.

### 2.6. Statistical Analysis

The statistical analysis for the evolution of the birth rate, perinatal mortality, and its fetal and early neonatal components was conducted via the calculation of rates and percentage rates. The birth rate (BR) was calculated for each year from 2008 to 2017 based on the ratio of the number of live births multiplied by 1000 (one thousand) per year and then divided by the total resident population, for which the 2010 census of the Brazilian Institute of Geography and Statistics (IBGE) was used.

The fetal mortality rate (FMR) was obtained via calculating the number of fetal deaths from 22 complete weeks of gestation (154 days) plus fetal deaths with an unknown or unfilled gestational age, of resident mothers, multiplied by 1000 (one thousand) and divided by the number of total births (live births plus fetal deaths after 22 weeks and more than gestation plus fetal deaths with an unknown or unfilled gestational age) of resident mothers [21]. The early neonatal mortality rate (ENMR) was obtained by calculating the number of deaths of residents aged 0 to 6 days multiplied by 1000 (one thousand) and divided by the number of live births of resident mothers [21]. Besides, the calculation of the perinatal mortality rate (PMR) was the sum of the number of fetal deaths from 22 weeks of gestation, plus fetal deaths with an unknown or unfilled gestational age, and deaths of children from 0 to 6 complete days of age from residents, multiplied by 1000 (one thousand) and divided by the total number of births to resident mothers (live births plus fetal deaths from 22 weeks onwards and more than gestation plus fetal deaths with unknown or unfilled gestational age) [21]. Calculations of perinatal mortality rates and their fetal and perinatal components were performed per year, and PMR, FMR and ENMR were calculated by category by variable, and the reference population was total births for PMR and FMR, and live births for ENMR.

To describe the variation that occurred in the response variable (Y) as a function of the set of explanatory variables (X), a simple linear regression model was applied in the analysis to quantify the relationship, and a simple linear regression calculator that uses the least-squares method was used to find the line of best fit for a set of paired data, described by the equation ŷ = bX + a, where b is the slope of the line and a is the intercept (i.e., the value of Y when X = 0). Data were added paired, one value per line, with the independent variable in the X values box and the dependent variable in the Y values box, accessed at https://www.socscistatistics.com/tests/regression/default.aspx, accessed on 29 August 2020. To calculate the mean predicted values in the spatial regression models with a square root transformation of the dependent variable, the linear regression analysis function available in Microsoft Excel version 2016 was used. The rating was very strong if it was greater or less than 0.9; strong if it was 0.7 to 0.9, positive or negative; and moderate if it was 0.5 to 0.7, positive or negative. The angular coefficient (β) and respective probability (p); prediction; and coefficient of determination (R^2^) were determined considering a significance level of α = 0.05.

To confirm the general trend of the PMR time series, a general volume and/or balance of volume (OBV) analysis was built, regarding the variations in the increase and decrease points of the perinatal mortality rate. For that, the PMR of 2008 was determined for the beginning of the calculation, from which we subtracted the value of the PMR presented in 2009 (the most current year), and so on, year by year, until 2017.

Considering possible events due to an exposure factor, the relative risk (RR) was calculated to measure its effect on perinatal death outcomes as well as the statistical significance of association or non-association, using the chi-square test (X^2^) for a 2 × 2 contingency table. The RR was expressed by the ratio between the absolute risks associated with the respective levels of the exposure variable considered, taking one of the levels as a reference, expressing the hypothesis null, considering that the reference level follows a normal distribution where the RR is equal to 1 (one). Therefore, the levels of each variable referring to the newborn’s, maternal, pregnancy, and childbirth characteristics were verified to observe the effect on the outcome. In this, levels of the variable with a certain number of elements (n) were selected, the RR was calculated to compare with the value of the null hypothesis. Considering the significance level, α = 0.05.

To assess the causes of perinatal death, the International Statistical Classification of Diseases and Related Health Problems (ICD-10) was adopted as a reference. Table 5 was prepared, which included its chapters, categories, codes, frequency, and percentage rates. Data processing was performed using Microsoft Office Excel® 2019 for Windows® for descriptive statistics, and the free statistical calculators on the Social Sciences Statistics website (https://www.socscistatistics.com/) were used for establishing correlations, accessed on 20 September 2019.

### 2.7. Ethical and Legal Aspects of This Research

This study complied with the ethical precepts guaranteed in the Declaration of Helsinki and in the Brazilian resolutions 466/12 and 510/16 of the National Health Council, which deal with research on human beings, and was approved by the Research Ethics Committee of the Santa Casa de Misericórdia de Vitória (EMESCAM), under protocol No. 2,738,639.

This research was funded by the Foundation for Research and Innovation Support of Espírito Santo (FAPES), under the notice FAPES/CNPq/Decit-SCTIE-MS/SESA nº. 03/2018—PPSUS.

## 3. Results

Of the 542,802 total births that occurred in the state of Espírito Santo, Brazil, 537,863 were live births, and 4939 were stillbirths (fetal deaths) while there were 3193 early neonatal deaths, totaling 8132 perinatal deaths (Table 1), between 2008 and 2017. The mortality rate perinatal was 15.0 per thousand births in the decade. The fetal/early neonatal ratio was 1.5:1. The percentage rate of fetal and early neonatal mortality in 10 years was 60.7% and 39.2%, respectively, among perinatal deaths; the fetal mortality rate (FMR) was 9.1/100,000 total births, and the early neonatal mortality rate (ENMR) was 5.9/1,000,000 live births (Table 1).

**Figure 1:**
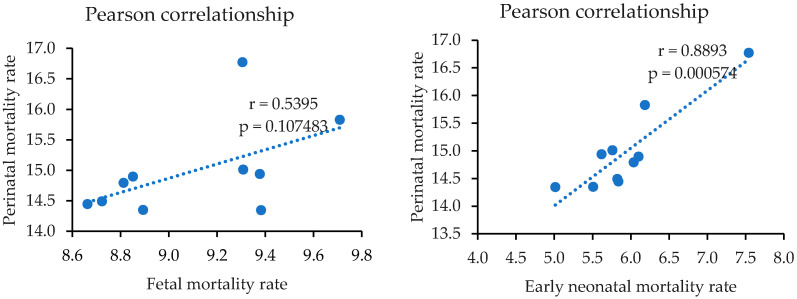
Effect of variations in fetal and early neonatal mortality rates on perinatal mortality rates. Espírito Santo, Brazil, 2008–2017. Source: MS/SVS/CGIAE—Sistema de Informações sobre Mortalidade (SIM) e MS/SVS/DASIS—Sistema de Informações sobre Nascidos Vivos (SINASC), 2021.

Figure 1 shows the effects of variations in fetal and early neonatal mortality rates on perinatal mortality rates. A positive correlation between FMR, ENMR, and PMR is assumed. It is also possible to perceive the strength of association between them by observing the proximity of the points compared to a straight line that cuts, as much as possible, through the center of the cloud of points. Considering the significance level, alpha 0.05, the result is non-significant in the Pearson correlation coefficient (r) of FMR, PMR, with a standard error = 0.6853; there was a strong positive correlation for ENMR, PMR in the simple linear regression equation: ŷ = 1.04 (95% CI 0.60; 1.43) X + 8.80, r (9) = 0.8893, with a significance level of *p* = 0.000574 and a standard deviation = 0.3723. This means that high scores of variable X go with high scores of variable Y (and vice versa).

**Figure 2:**
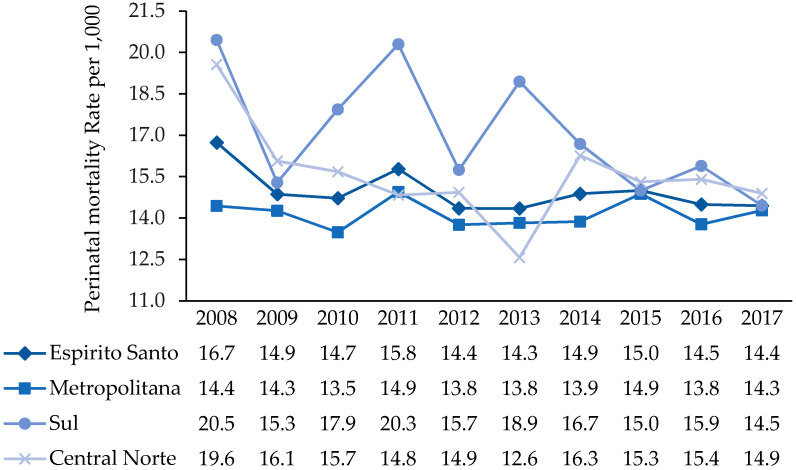
Perinatal mortality rate by health region. Espírito Santo, Brazil, 2008–2017. **Source**: MS/SVS/CGIAE—Sistema de Informações sobre Mortalidade (SIM) e MS/SVS/DASIS—Sistema de Informações sobre Nascidos Vivos (SINASC), 2021.

Figure 2 shows the results of the analyses performed on the behavior of the perinatal mortality rate by health region and in Espírito Santo between 2008 and 2017. The results show a decreasing trend in the PMR in Espírito Santo, from 16.8/1000 in 2008 to 14.4/1000 in 2017 (Table 1 and Figure 2), a reduction of around 14.3% in 10 years and 1.4% per year. From 2008 to 2017, the early neonatal component (zero to six full days of life) showed a reduction of 22.7%, and the fetal component showed a smaller decrease of 6.5%.

In the analysis by health region, differences in results by region were observed. From 2008 to 2017, the South region had a PMR of about 17.1/1000 total births, while the Central North and Metropolitan regions had the lowest average rates (15.6 and 14.2/1000 total births). The greatest reduction in the PMR was observed in the health region of the South, around 29.3%, followed by the Central North, 23.9%. Additionally, the smallest reduction was presented by the Metropolitan region, 1.1% (Figure 2). In 2017, the Central North region had the highest PMR, 14.9/1000 total births.

Between 2008 and 2017, the trend analysis showed cyclical variations in the PMR, from peaks to peaks and troughs, although there was an evident downward trend. Among the health regions, irregular variations were frequent and intense in the South region, followed by the Central North. The South region, compared to other health regions, had the lowest presence of high and low peaks (Figure 2).

Regarding the 78 municipalities that make up the health regions, 89.74% (70 municipalities) recorded up to 139 fetal deaths and 10.26% (eight municipalities) recorded a range of 139 to 684 fetal deaths. The data for early neonatal deaths show that 88.46% (69) of the municipalities recorded up to 82 cases and that 11.54% (nine municipalities) recorded a range of 82 to 402 cases. The highest absolute frequency of early fetal and neonatal deaths was concentrated in the municipalities of Serra, Cariacica, and Vila Velha, accompanied by Cachoeiro de Itapemirim, Guarapari, Linhares, São Mateus, Vitória, and Colatina.

**Figure 3:**
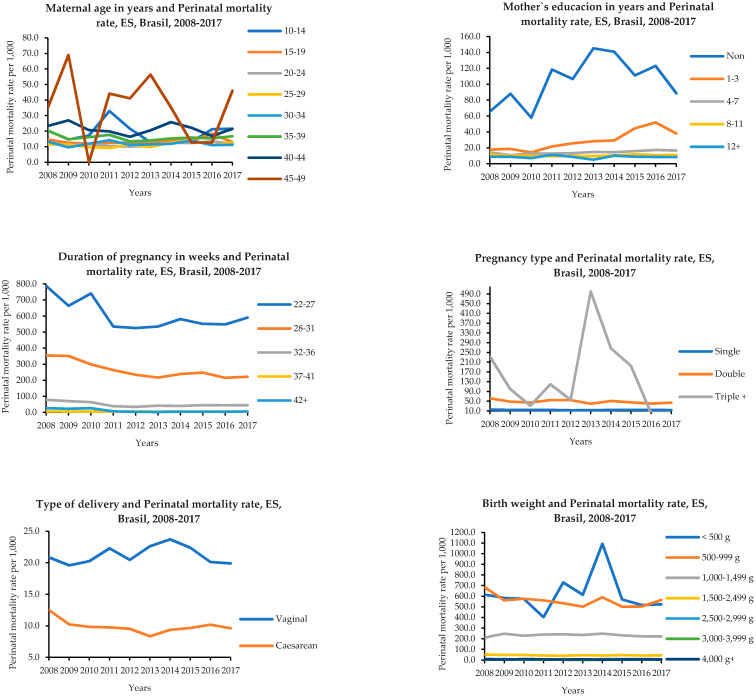
Factors associated with the trend in perinatal mortality. Espírito Santo, Brazil, 2008–2017. **Source**: MS/SVS/CGIAE—Sistema de Informações sobre Mortalidade (SIM) e DASIS—Sistema de Informações sobre Nascidos Vivos (SINASC), 2021.

When analyzing perinatal deaths and their early fetal and neonatal components by maternal characteristics, pregnancy, childbirth, and place of occurrence, as shown in Table 2 and Figure 3, the following was found: from 2008 to 2017, 65.1% of perinatal deaths were related to mothers between 20 and 39 years of age, an occurrence that extended to the early fetal and neonatal intervals. However, it can be observed that mothers aged between 40 and 49 years had a higher PMR, 22.1/1000, and between 10 and 19 years old, 13.8/1000, a situation that was repeated with the FMR and ENMR, respectively, 15.8; 7.6; 6.3; and 6.2/1000.

The analysis of the education of mothers showed that 59.1% of perinatal deaths were related to those with four to eleven years of schooling. However, the perinatal mortality rate per thousand births among mothers without any year of schooling prevailed, at 96.5/1000. This also extends to the fetal components, the FMR, 55.9/1000, and the ENMR, 42.9/1000 (Table 2 and Figure 3).

The observation of the fetal age or gestational age factor revealed that 66.4% of perinatal deaths occurred in premature conditions, 23.7% in term, and 0.7% post-term, and the highest percentage of perinatal deaths, 23.7%, was concentrated in the gestational interval from the 22nd to the 27th week of gestation, corresponding to a PMR of 605.0/1000 (Table 2 and Figure 3).

Perinatal death was more common in the triplet pregnancy, 133.9/1000, a higher rate than in the double pregnancy, 47.7/1000. The lowest rate was related to single pregnancy, 13.4/1000. There was a higher rate of perinatal deaths related to vaginal delivery, 21.2/1000, compared to cesarean delivery, 9.9/1000. As for the place of occurrence of perinatal death, the household, with a rate of 191.7/1000, exceeded the other places of occurrence, such as another health establishment, 28.4/1000, and a hospital, 14.3/1000 (Table 2 and Figure 3).

The study did not propose to perform an incomplete analysis of the completion of notifications of perinatal deaths, category ignored or not filled out. For this reason, we express their absolute values and percentage rates by the following variables: maternal age, 1536 (15.8%), maternal education, 1905 (23.4%), duration of pregnancy, 753 (9.3%), type of pregnancy, 466 (5.7%), type of delivery, 508 (6.2%), place of occurrence, 41 (0.5%), sex, 279 (3.4%), race/skin color, 5.328 (65.5%), and birth weight, 1.255 (15.4%) (Table 2 and Table 3).

In the analysis of birth weight and perinatal mortality, a higher risk for mortality was observed for a birth weight less than 500 g, 593.4/1000, followed by 500 g to 999 g, 556.3/1000. Although the brown and white races had the highest percentage rates (13.6% and 19.8%, respectively), the rates per thousand total births were concentrated in the white and indigenous races/skin colors (6.8 and 6.2/1000, respectively). From 2008 to 2017, 52.9% of perinatal deaths were male, a PMR of 15.5/1000, a condition that was repeated in the fetal and early neonatal intervals (Table 3 and Figure 3).

There was a strong association between perinatal mortality and the place of occurrence being a hospital, r (9) = 0.9665, *p* ≤ 0.00001; male sex, r (9) = 0.7674, *p* = 0.010; race/color of black, r (9) = 0.669, *p* = 0.034 and brown, r (9) = 0.6985, *p* = 0.025; single pregnancy, r (9) = 0.8825, *p* = 0.001; cesarean delivery, r (9) = 0.8061, *p* = 0.005; birth weight ≥ 2500, r (9) = 0.8072, *p* = 0.005; a gestational age of 37–41 weeks, r (9) = 0.8503, *p* = 0.002; and a moderate one with double pregnancy, r (9) = 0.789, *p* = 0.007.

In Table 4, by adopting the statistical method of measuring the effects of each of the factors assumed by each level of characteristics (variables) of perinatal deaths and their early fetal and neonatal components, it was observed that the risk of death in the perinatal period male group is about 1.15 times the risk of those in the female group (95% CI, 1.10:1.20). In triple pregnancy it is about 10.0 (95% CI, 6.98:14.31) times the risk than in single pregnancy, and it is about 3.56 (95% CI, 3.25:3.90) times more in double pregnancy compared to a single pregnancy. Taking a birth weight ≥ 2500 g as a reference, at a birth weight < 1500 g the risk is about 99.38 (95% CI, 93.58:105.54) times more, followed by a birth weight between 1500 g and 2.499 g, RR = 11.54 (95 CI%, 10.80:12.33). Using mothers aged between 20 and 29 years as a reference, mothers aged < 19 years contributed about 1.20 (95% CI, 1.12:1.28) times more to the occurrence of perinatal death, followed by mothers aged ≥ 29, RR = 1.20 (95% CI, 1.14:1.27). Using mothers with ≥12 years of schooling as a reference, mothers without schooling had a risk of about 11.44 (95% CI, 9.76:13.41) times more, those with 1 to 3 years of schooling had a risk of 2.96 (95% CI, 2.22:3.34) times more, those with 4 to 7 years of education had a risk of 1.67 (95% CI, 1.53:1.81) times more, and mothers with 8–11 years of education had a risk of 1.24 (95% CI, 1.15:1.34) times more. The risk related to the place of occurrence being at home was 13.40 (95% CI, 11.30:15.88) times higher than in a hospital.

The analysis of the groups of causes of perinatal deaths between 2008 and 2017, based on the chapters and categories of the ICD-10, indicates that among the perinatal conditions those referring to the fetus and newborn affected by maternal factors and complications of pregnancy, labor, and delivery appeared as the first cause, 49.70%, followed by intrauterine hypoxia and birth asphyxia, 11.03%, followed by other respiratory disorders originating in the perinatal period, 6.48%. Among the causes, avoidable ones predominate, reducible by adequate care for the fetus, the pregnant woman, the childbirth, and the newborn. For the causes of death, 0.61% were represented by “some infectious and parasitic diseases”, and 13.02% were represented by “congenital malformations, deformities, and other congenital anomalies” (Table 5).

**Figure 4:**
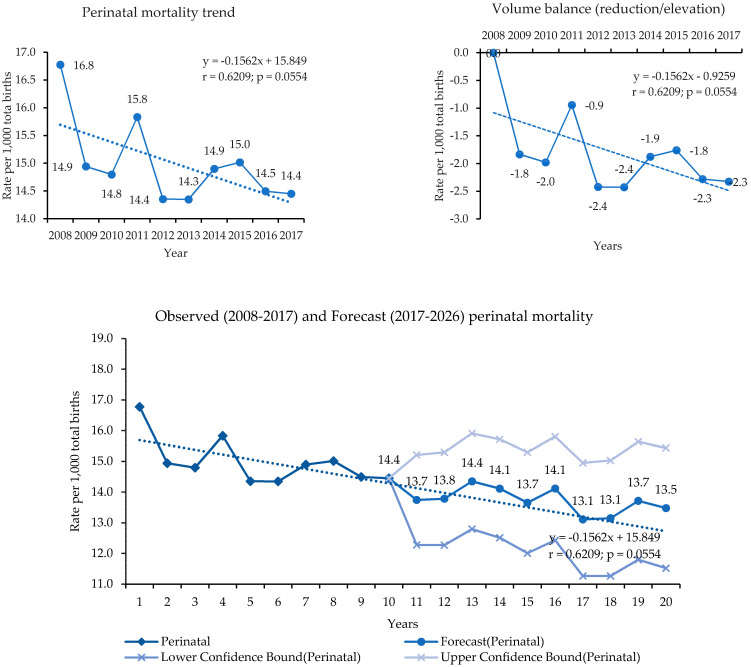
Observed and predicted perinatal mortality and volume balance. Espírito Santo, Brazil, 2008–2017. **Source**: MS/SVS/CGIAE—Sistema de Informações sobre Mortalidade (SIM) e DASIS—Sistema de Informações sobre Nascidos Vivos (SINASC), 2021.

Regarding the observed and predicted PMR, the observed trend was decreasing for stationary behavior, with a negative slope (β1 = −0.1562). The volume balance (OBV) was cumulative, composed of falls and rises, with a final volume of negative 2.3. The OBV confirmed a slow (stationary) decreasing trend, with a negative result of 0.23 per year (Figure 4).

Along with the trend, there were irregular variations, with high points in 2011, 2014, and 2015, and low points in 2009, 2010, 2012, and 2013, without characterizing sudden changes. The series has a particular behavior over time, indicating that it has a high probability of following this same behavior for the next 10 years (Figure 4).

## 4. Discussion

The decline in perinatal mortality in the state of Espírito Santo, Brazil, and in health regions was not significant between 2008 and 2017. In the 10 years analyzed by this study, the reduction in perinatal, fetal, and neonatal mortality in the state corresponded to 14.3%, 6.5%, and 22.7%, respectively. The total reduction was two deaths per thousand total births in the period, demonstrating an annual drop of about 0.23/1000 total births, less than one death each year.

Several factors contributed to the reduction in perinatal mortality, such as the expansion of beds, the quality of neonatal care, campaigns to encourage breastfeeding, improvement of prenatal care and childbirth, and the implementation of Rede Cegonha, in addition to the expansion of programs such as the Bolsa Família, Family Health Strategy, and Mais Médicos [22,23]. However, reductions in perinatal and infant mortality are linked to other conditions, some of which are maternal factors, such as age and parity [24], which can influence birth weight, the main determinant of survival [25,26].

Concerning maternal conditions, it was observed that mothers’ ages ranging from 10 to 19 years old and 40 to 49 years old were the ones that most influenced the perinatal mortality rates. However, this is not the factor to which the reductions in perinatal mortality that have occurred can be attributed. Explanations for the decreases can be improvements in prenatal care, delivery, and newborn care, considering that from 1990 to 2015 efforts were directed towards reducing neonatal deaths in the perinatal interval, and the variation in values most influenced the variations in PMR between 2008 and 2017, which also had a greater reduction, observed in this study.

Measures to reduce stillbirths received less attention and investment, as they were not specifically addressed in the Millennium Development Goals (MDGs) from 2000 to 2015 and only started with the Sustainable Development Goals (SDGs) [9] from 2015, which go until 2030, evidence that helps to explain the current position of the reduction in fetal mortality rates, and which also draws attention to the urgency of developing actions and services with goals that focus on correcting the observed values. The World Health Assembly, which monitors the health indicators of the SDGs, set a goal in the Plan of Action that by 2030 all countries will have to achieve a rate of 12 or fewer stillbirths per 1000 total births [27]. According to this goal, the state of Espírito Santo has been achieving what is being estimated, needing only to overcome other factors that influence stillbirth rates.

Assertive actions begin by reducing the differences in perinatal mortality between the Health Regions of the state of Espírito Santo. Regional differences persisted throughout the decade of the study, and in the computation influenced the index of the state perinatal indicator. This is a situation that requires effort and adequate strategies, since differences in perinatal mortality rates are not just a local event as they are observed between continents, countries, and regions of the world with the presence of high and low rates. It should also be noted that there is a difference in the variation in the perinatal mortality rate between the regions of the state of Espírito Santo, which may be due to their development. The Metropolitan region is the most populous in the state; however, it has more health services prepared to meet this demand, while the South region of the state, which had the highest mortality rate, is also quite populous, but has few health services [22,23,24,25,26].

Among the high rates are Ethiopia (46/1000 births) [28] and the Democratic Republic of Congo, also in Africa (>40/1000) [29]. The United States has a rate equal to 6.24/1000 in the year 2013 [6], and England and Wales had a coefficient equal to 6.5/1000 in 2015 [30]. South America has Chile, with a rate of 12.6/1000 [31] in 2010, and the city of Manizales, in Colombia, had an average rate of 9.7/1000 between the years 2009 and 2012 [31]. It is noteworthy that, in these international studies, the calculation of perinatal mortality starts in the 28th week of pregnancy, and not in the 22nd week, as is the case for most studies carried out in Brazil, such as the present work. In Brazil, in 2017, Espírito Santo reached a perinatal mortality rate of 14.4 deaths per thousand births. In 2010, Maranhão (38.5/1000) [32] and Alagoas (35.2/1000) were the Brazilian states with the highest perinatal mortality rates [33]. The states with the lowest rates were Rio Grande do Sul (13.7/1000), São Paulo (13.6/1000), and Santa Catarina (12.8/1000) in 2011 [31], in which the rate found in this study for the state of Espírito Santo was higher, with a value of 15.0 per total births.

In a study carried out in Pernambuco, Brazil, between 2010 and 2014, a perinatal mortality rate of 15.3 per thousand total births was observed, corroborating the findings of this study. However, the values of components, such as fetal deaths (8.9 deaths per thousand births), and early neonatal deaths (6.5 deaths per thousand live births) [34], are divergent. Additionally, when evaluating risk factors for perinatal death in a cohort in the city of Pelotas, Rio Grande Sul (Southern Brazil), they identified a higher PMR (22.1/1000 births), a value much higher than that of the present study [34].

In Espírito Santo, the fetal mortality rate stands out, which despite the low percentage of reduction found in this study, the result is lower than that presented by Brazil (10.0 per 1000 births) and the Northeast Region (12.1 per 1000 births); regardless, it is high compared to developed countries (2 to 7 per 1000 births) [35]. In 1949 stillbirths were added to early neonatal deaths to create the perinatal mortality indicator, being fundamental to evaluate the perinatal indicator by exploring its components [36]. In the state, between 2008 and 2017, the number of deaths in the fetal range was 35.5% higher than in the early neonatal range, and the reduction in deaths in the early neonatal component was higher by 62.30%, compared to the fetal component. Differences in perinatal mortality rates were better perceived in studies addressing regional disparities [37].

It was also noted that, when comparing the records of fetal and early neonatal deaths, there was a greater proportion of ignored information for fetal deaths. A systematic review of fetal deaths in Brazil pointed out that there was an improvement in filling out the death certificate, but there are gaps in regard to the filling out of sociodemographic variables in particular [36]. In this way, inaccuracies are shown in the underlying causes of deaths, through investigations carried out by death surveillance [38].

The audit and review of perinatal death, as a tool, probably improves the quality of care and the incompleteness in filling out the death notification, being a WHO recommendation for the assessment of maternal and perinatal deaths in all hospitals worldwide [39]. The analysis of maternal and perinatal deaths has its importance in the epidemiological control of and reduction in morbidity and mortality, and in its genesis it systematizes the characteristics of deaths, considering different relationships, being an indicator of the quality of healthcare for women and children [40,41,42].

These characteristics, if maternally related to the middle age range for women to be pregnant with lower socioeconomic levels and a low level of education, perinatal deaths persist [43]. There are identified factors related to perinatal mortality, which can be poor obstetric history, a short interpartum interval, multiple pregnancies, a history of stillbirth, hypertension, diabetes, lack of prenatal care, and low socioeconomic status [44]. Increased risk of perinatal death was associated with a low level of maternal education, rural residence, <4 ANC visits, PPH, placental abruption, LBW delivery, the child’s sex, and referral for delivery [45]. Prematurity, a low birth weight, breech presentation, multiple gestation, and cesarean delivery reduce preventability goals related to perinatal death [46].

These data and the approach used to acquire them can be used to inform interventions aimed at reducing the rate of stillbirths and neonatal deaths in non-developed, developing, and developed countries that, even with low perinatal mortality rates, are influenced by factors that characterize the deaths [13,46,47,48].

This study on Espírito Santo highlighted the biological aspects of newborns and fetuses, prematurity and low birth weight, considered to be the main predictors for perinatal mortality, and extreme prematurity is a relevant risk factor as well as being associated with a lower gestational age [48,49,50,51]. However, other aspects can influence perinatal mortality, such as maternal health status, access to and quality of health services, socioeconomic status, and the environment in which they live [50].

There was a predominance of perinatal death through vaginal delivery; this route is natural and preferred for births in general. Additionally, specifically, in fetal cases, vaginal delivery is the recommended route [13,52]. One study pointed out that in the reduction in perinatal mortality there is no direct relationship with the type of delivery, but rather with the improvement of perinatal care [53].

Regarding the perinatal deaths observed in Espírito Santo from 2008 to 2017, according to the List of Causes of preventable deaths by the Unified Health System (SUS) [54], about 86% were preventable [55]. Among the preventable causes, those that can be reduced by adequate care for newborns predominate, including the situation of prematurity [56,57,58]. However, preventable prematurity is very often related to the quality of prenatal care [14,50,59,60]. The magnitude of the causes related to care during pregnancy, childbirth, and the newborn demonstrate the importance of prenatal and childbirth care in reducing child mortality in Brazil [54,55,61].

Consolidating the organization of perinatal care in Espírito Santo is essential for the prompt recognition of risk situations, surveillance, and health promotion, considering the potential observed in providing a positive response in the control of perinatal mortality, but with a result of a not very significant volume, indicating that it has a high probability of following this same behavior for the next 10 years. The Brazilian Ministry of Health recognized the slow achievement of goals for reducing perinatal and infant mortality, and in 2016 created a synthesis of evidence for health policies on perinatal mortality with the following proposals: (1)—clinical protocol for prenatal care; (2)—increase the time interval between pregnancies; (3)—presence of a companion for early and continuous support to the pregnant woman during labor; and (4)—the use of corticosteroids to prevent respiratory distress in premature infants [55].

In a systematic review of perinatal mortality in the world context, the following recommendations were given: training teams in neonatal resuscitation; late clamping of the umbilical cord; sustainable financing mechanisms to provide integrated, high-quality services in prenatal, delivery, and postpartum care; the use of nutritional education during prenatal care; screening and treating genitourinary tract infections; qualified assistance at the time of delivery; and the need for investments and strategies to achieve greater equity [62]. However, as SUS establishes equal opportunity of access to healthcare for equal needs, there is inequality in supply and structural resources, associated with inadequate and untimely healthcare, with significant implications for perinatal mortality.

The limitations present in this work should be highlighted, namely: the incomplete-ness of some variables; the problems of classifying neonatal deaths that are diagnosed as stillborn; incorrect filling of the DO; under-registration; and a lack of information, which can contribute to the underestimation of rates. There is a need for the evaluation and reformulation of perinatal healthcare policies, intending to improve the performance and quality of care provided and healthcare for the mother–child binomial, in reproductive planning, prenatal care, and in care during childbirth and puerperium as well as to the newborn.

## 5. Conclusions

The analysis showed a slow downward trend in perinatal mortality with peaks of highs and lows, with a reduction and elevation result (volume balance) of negative 2.3, in the decade. Differences were observed in trends between health regions, constancy of risk factors related to adolescent and elderly mothers, preterm birth, twin pregnancy, and low birth weight, risks that are similar to Brazilian and international states. The majority of perinatal deaths were reducible by adequate care for women during pregnancy and childbirth as well as for fetuses and newborns, concentrated on causes related to the fetuses and newborns being affected by maternal factors and complications of pregnancy, duck labor, childbirth, intrauterine hypoxia, and birth asphyxia.

## Figures and Tables

**Table 1:** Perinatal, fetal, and early neonatal mortality rates, birth rates, and percentage rates. Espírito Santo, Brazil, 2008–2017.

Years	Natality			Mortality (per 1000)								
	Live Births			Fetal			Early Neonatal			Perinatal		
	f	%	R	f	%*	R	f	%*	R	f	%	R
2008	51,852	9.6	14.8	487	55.5	9.3	391	44.5	7.5	878	10.8	16.8
2009	51,457	9.6	14.6	487	62.8	9.4	289	37.2	5.6	776	9.5	14.9
2010	51,853	9.6	14.8	461	59.6	8.8	313	40.4	6.0	774	9.5	14.8
2011	53,053	9.9	15.1	520	61.3	9.7	328	38.7	6.2	848	10.4	15.8
2012	52,835	9.8	15.0	474	62.0	8.9	291	38.0	5.5	765	9.4	14.4
2013	54,065	10.1	15.4	512	65.4	9.4	271	34.6	5.0	783	9.6	14.3
2014	56,548	10.5	16.1	505	59.4	8.9	345	40.6	6.1	850	10.5	14.9
2015	56,941	10.6	16.2	535	62.0	9.3	328	38.0	5.8	863	10.6	15.0
2016	53,413	9.9	15.2	470	60.2	8.7	311	39.8	5.8	781	9.6	14.5
2017	55,846	10.4	15.9	488	60.0	8.7	326	40.0	5.8	814	10.0	14.4
Total	537,863	100.0	153.0	4.939	60.7	9.1	3.193	39.3	5.9	8132	100.0	15.0

**Source**: MS/SVS/CGIAE—Sistema de Informações sobre Mortalidade (SIM) e MS/SVS/DASIS—Sistema de Informações sobre Nascidos Vivos (SINASC), 2021. **Note**: Frequency (f). Percentage of fetal and early neonatal death in the composition of perinatal death, per year and within 10 years (%*). Percentage rate (%). Rate (R). Ministério da Saúde (MS). Secretaria de Vigilância em Saúde (SVS). Coordenação-Geral de Informações e Análises Epidemiológicas (CGIAE). Diretoria de Apoio ao Sistema de Saúde (DASIS).

**Table 2:** Perinatal, fetal, and early neonatal mortality by maternal characteristics, pregnancy, childbirth, and place of occurrence. Espírito Santo, Brazil, 2008–2017.

Characteristics		Mortality Rates per Thousand and Proportional (%)								
		Fetal			Early Neonatal			Perinatal		
	LBs	f	FMR	%	f	ENMR	%	f	PMR	%
Mother’s age (years)										
10 to 19	92,862	707	7.6	14.3	583	6.2	18.3	1290	13.8	15.9
20 to 29	273,829	1875	6.8	38.0	1296	4.7	40.6	3171	11.5	39.0
30 to 39	159,129	1333	8.3	27.0	787	4.9	24.6	2120	13.2	26.1
40 to 49	11,988	193	15.8	3.9	76	6.3	2.4	269	22.1	3.3
50+	52	0	0.0	0.0	1	19.2	0.0	1	19.2	0.0
Ignored	3	831	996.4	16.8	450	150,000	14.1	1281	153.6	15.8
Mother’s education (years)										
Non	2026	120	55.9	2.4	87	42.9	2.7	207	96.5	2.5
1 to 3	15,861	272	16.9	5.5	130	8.2	4.1	402	24.9	4.9
4 to 7	122,975	1087	8.8	22.0	657	5.3	20.6	1744	14.1	21.4
8 to 11	290,732	1796	6.1	36.4	1269	4.4	39.7	3065	10.5	37.7
12+	95,399	451	4.7	9.1	357	3.7	11.2	808	8.4	9.9
Ignored	10,870	1212	100.3	24.5	693	63.8	21.7	1905	157.7	23.4
Duration of pregnancy (weeks)										
22 to 27	2356	1054	309.1	21.3	1009	428.3	31.6	2063	605.0	25.4
28 to 31	4599	941	169.9	19.1	482	104.8	15.1	1423	256.9	17.5
32 to 36	39,755	1348	32.8	27.3	565	14.2	17.7	1913	46.5	23.5
37 to 41	470,370	1168	2.5	23.6	756	1.6	23.7	1924	4.1	23.7
42+	10,428	30	2.9	0.6	26	2.5	0.8	56	5.4	0.7
Ignored	10,355	398	37.0	8.1	355	34.3	11.1	753	70	9.3
Pregnancy type										
Single	526,338	4467	8.4	90.4	2641	5.0	82.7	7108	13.4	87.4
Double	10,748	243	22.1	4.9	281	26.1	8.8	524	47.7	6.4
Triple/more	237	17	66.9	0.3	17	71.7	0.5	34	133.9	0.4
Ignored	540	212	281.9	4.3	254	470.4	8.0	466	619.7	5.7
Delivery										
Vaginal	199,065	2917	14.4	59.1	1359	6.8	42.6	4276	21.2	52.6
Caesarean	338,081	1788	5.3	36.2	1560	4.6	48.9	3348	9.9	41.2
Ignored	717	234	246.1	4.7	274	382.1	8.6	508	534.2	6.2
Place of occurrence										
Hospital	529,450	4537	8.5	91.9	3101	5.9	97.1	7638	14.3	93.9
Other health centers	7347	177	23.5	3.6	37	5.0	1.2	214	28.4	2.6
Home	701	139	165.5	2.8	22	31.4	0.7	161	191.7	2.0
Public highway	0	11	1000	0.2	12	0.0	0.4	23	2090.9	0.3
Other	341	39	102.6	0.8	16	46.9	0.5	55	144.7	0.7
Ignored	24	36	600.0	0.7	5	208.3	0.2	41	683.3	0.5
**Total**	**537,863**	**4939**	**9.1**	**100.0**	**3193**	**5.9**	**100.0**	**8132**	**15** **.0**	**100.0**

**Source**: MS/SVS/CGIAE—Sistema de Informações sobre Mortalidade (SIM) e MS/SVS/DASIS—Sistema de Informações sobre Nascidos Vivos (SINASC), 2021. Note: Live births (LBs). Frequency (f). Percentage rate (%). Perinatal mortality rate (PMR). Fetal mortality rate (FMR). Early neonatal mortality rate (ENMR). Ministério da Saúde (MS). Secretaria de Vigilância em Saúde (SVS). Coordenação-Geral de Informações e Análises Epidemiológicas (CGIAE). Diretoria de Apoio ao Sistema de Saúde (DASIS).

**Table 3:** Perinatal, fetal, and early neonatal mortality due to the characteristics of the stillbirth and the newborn. Espírito Santo, Brazil, 2008–2017.

Characteristics		Mortality Rate per Thousand and Proportional (%)								
(Fetus and Newborn)		Fetal			Early Neonatal			Perinatal		
	LBs	f	FMR	%*	f	ENMR	%*	f	PMR	%
Sex										
Male	276,029	2521	9.1	51.0	1783	6.5	55.8	4304	15.5	52.9
Female	261,773	2169	8.2	43.9	1380	5.3	43.2	3549	13.4	43.6
Ignored	61	249	803.2	5.0	30	491.8	0.9	279	900.0	3.4
Color/race										
White	162,982	39	0.2	0.8	1067	6.5	33.4	1106	6.8	13.6
Black	21,801	13	0.6	0.3	61	2.8	1.9	74	3.4	0.9
Yellow	829	0	0.0	0.0	5	6.0	0.2	5	6.0	0.1
Mixed	336,260	130	0.4	2.6	1481	4.4	46.4	1611	4.8	19.8
Indigenous	1169	0	0.0	0.0	8	6.8	0.3	8	6.2	0.1
Ignored	14,822	4757	243.0	96.3	571	38.5	17.9	5328	272.1	65.5
Birth weight										
<500 g	436	142	245.7	2.9	201	461.0	6.3	343	593.4	4.2
500 to 999 g	2392	998	294.4	20.2	888	371.2	27.8	1886	556.3	23.2
1000 to 1499 g	3865	655	144.9	13.3	398	103.0	12.5	1053	233.0	12.9
1500 to 2499 g	35,956	1104	29.8	22.4	560	15.6	17.5	1664	44.9	20.5
2500 to 2999 g	114,449	485	4.2	9.8	329	2.9	10.3	814	7.1	10.0
3000 to 3999 g	349,382	525	1.5	10.6	424	1.2	13.3	949	2.7	11.7
4000 g+	31,343	111	3.5	2.2	57	1.8	1.8	168	5.3	2.1
Ignored	40	919	958.3	18.6	336	8400.0	10.5	1255	1308.7	15.4
Total	537,863	4939	9.1	100.0	3193	5.9	100.0	8132	15.0	100.0

**Source**: MS/SVS/CGIAE—Sistema de Informações sobre Mortalidade (SIM) e MS/SVS/DASIS—Sistema de Informações sobre Nascidos Vivos (SINASC), 2021. **Note**: Live births (LBs). Percentage of fetal and early neonatal death in the composition of perinatal death, per year and within 10 years (%*). Percentage rate (%). Rate (R). Perinatal mortality rate (PMR). Fetal mortality rate (FMR). Early neonatal mortality rate (ENMR). Ministério da Saúde (MS). Secretaria de Vigilância em Saúde (SVS). Coordenação-Geral de Informações e Análises Epidemiológicas (CGIAE). Diretoria de Apoio ao Sistema de Saúde (DASIS).

**Table 4:** Factors associated with risk of perinatal complications, according to maternal, pregnancy, and the newborn’s characteristics in the state of Espírito Santo, Brazil, 2008–2017.

Factors (NB, Pregnancy, Delivery, and Maternal) (NB, Maternal/Pregnancy)	2008–2017				
	PD	TBs	PMR	RR (95% CI)	*p*-Value
Sex					
Male	4304	278,550	15.5	1.15 (1.10:1.20)	<0.00001
Female	3549	263,942	13.4	1.00	(Reference)
Race/skin color					
Black	74	21,814	3.4	0.50 (0.39:0.63)	<0.00001
Mixed	1611	336,390	4.8	0.71 (0.65:0.76)	<0.00001
White	1106	163,021	6.8	1.00	(Reference)
Pregnancy type					
Double	524	10,991	47.7	3.56 (3.25:3.90)	<0.00001
Triple/more	34	254	133.9	10.00 (6.98:14.31)	<0.00001
Single	7108	530,805	13.4	1.00	(Reference)
Delivery type					
Cesarean	3348	339,869	9.9	0.47 (0.44:0.49)	<0.00001
Vaginal	4276	201,882	21.2	1.00	(Reference)
Birth weight (grams)					
<1.500	3282	8488	386.66	99.38 (93.58:105.54)	<0.00001
1.500 to 2.499	1664	37,060	44.90	11.54 (10.80:12.33)	<0.00001
≥2.500	1931	496,295	3.89	1.00	(Reference)
Gestational age (weeks)					
<37	5399	50,053	107.9	26.44 (25.08:27.87)	<0.00001
37–41	1924	471,538	4.1	1.00	(Reference)
≥42	56	10,458	5.4	1.31 (1.01:1.71)	<0.00001
Mother’s age (years)					
<19	1290	93,569	13.8	1.20 (1.12:1.28)	<0.00001
20–29	3171	275,704	11.5	1.00	(Reference)
≥29	2389	172,695	13.8	1.20 (1.14:1.27)	<0.00001
Maternal education (years)					
No education	207	2146	96.5	11.44 (9.76:13.41)	<0.00001
1–3	402	16,133	24.9	2.96 (2.62:3.34)	<0.00001
4–7	1744	124,062	14.1	1.67 (1.53:1.81)	<0.00001
8–11	3065	292,528	10.5	1.24 (1.15:1.34)	<0.00001
≥12	808	95,850	8.4	1.00	(Reference)
Place of occurrence					
Hospital	7638	533,987	14.3	1.00	(Reference)
Other health estab.	214	7524	28.4	1.99 (1.79:2.28)	<0.00001
Home	161	840	191.7	13.40 (11.30:15.88)	<0.00001

**Source**: MS/SVS/CGIAE—Sistema de Informações sobre Mortalidade (SIM) e MS/SVS/DASIS—Sistema de Informações sobre Nascidos Vivos (SINASC), 2021. Note: Newborn (NB). Perinatal death (PD). Total births (TBs). Perinatal mortality rate (PMR). Relative risk (RR). Confidence interval (CI). Ministério da Saúde (MS). Secretaria de Vigilância em Saúde (SVS). Coordenação-Geral de Informações e Análises Epidemiológicas (CGIAE). Diretoria de Apoio ao Sistema de Saúde (DASIS).

**Table 5:** Perinatal deaths by ICD-10 classification. Espírito Santo, Brazil, 2008–2017.

ICD-10th Chapters/Categories of Causes and Codes	Perinatal Mortality	
	f	%
**I. Some infectious and parasitic diseases**	**50**	**0.61**
Congenital syphilis (A50)	49	0.60
Other bacterial diseases (A21–A22, A24–A28, A31–A32, A38, and A42–A49)	1	0.01
**XVI. Some conditions originating in the perinatal period**	**6997**	**86.04**
Fetus and newborn affected by maternal factors and complications of pregnancy, labor, and delivery (P00–P04)	4042	49.70
Fetal growth retardation, fetal malnutrition, and disorders related to short gestation and low birth weight (P05–P07)	290	3.57
Trauma during birth (P10–P15)	12	0.15
Intrauterine hypoxia and birth asphyxia (P20–P21)	897	11.03
Other respiratory disorders originating in the perinatal period (P22–28)	527	6.48
Congenital infectious and parasitic diseases (P35–P37)	229	2.82
Other conditions originating in the perinatal period (P38–39, P55, P08, P29, P50–P54, and P56–P96)	1000	12.30
**XVII. Congenital malformations, deformities, and other congenital anomalies**	**1059**	**13.02**
Spina bifida (Q05)	6	0.07
Congenital malformations of the circulatory system (Q20–Q28)	234	2.88
Absence, atresia, and small bowel stenosis (Q41)	4	0.05
Other congenital malformations and deformities of the musculoskeletal system (Q67–Q79)	107	1.32
Other malformations, deformities, and other anomalies (Q00–Q04, Q06–Q07, Q10–Q18, Q30–Q34, Q35–Q37, Q50–Q52, Q54–Q64, Q80–Q89, and Q90–Q99)	708	8.71
… Other chapters and categories of causes	26	0.32

**Source**: MS/SVS/CGIAE—Sistema de Informações sobre Mortalidade (SIM) e MS/SVS/DASIS—Sistema de Informações sobre Nascidos Vivos (SINASC), 2021. **Note**: Frequency (f). Percentage rate (%). Ministério da Saúde (MS). Secretaria de Vigilância em Saúde (SVS). Coordenação-Geral de Informações e Análises Epidemiológicas (CGIAE). Diretoria de Apoio ao Sistema de Saúde (DASIS).

## Data Availability

All data were taken from public systems of the Federal Government of Brazil, through the Information Technology Department of the Brazilian Unified Health System (DATASUS), which can be accessed through the link, specifically in the SIM and SINASC systems: https://datasus.saude.gov.br/.
